# Supplementary material for: Multiparent-Derived, Marker-Assisted Introgression Lines of the Elite Indian Rice Cultivar, ‘Krishna Hamsa’ Show Resistance against Bacterial Blight and Blast and Tolerance to Drought
Source: Plants (Basel). 2022 Feb 25;11(5):622. doi: 10.3390/plants11050622 (PMC8912774; doi:10.3390/plants11050622)
Supplement: Supplementary file 1 [file plants-11-00622-s001.zip › plants-1577775-supplementary.pdf]

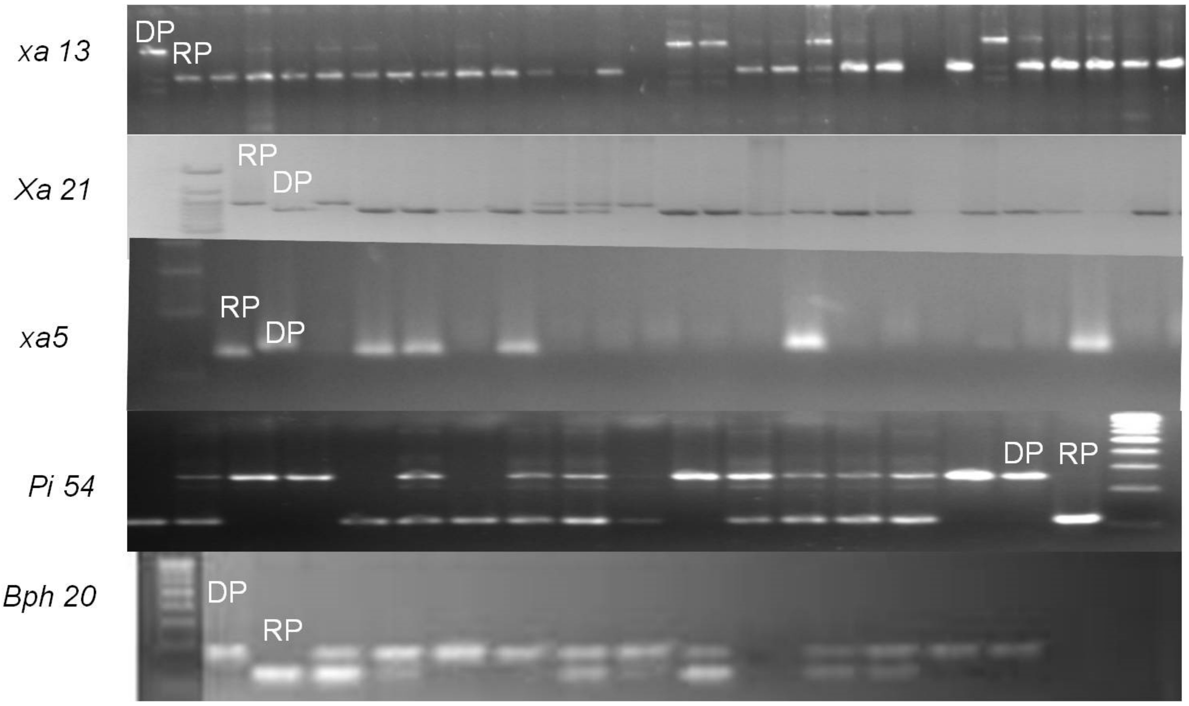

Supplementary Figure S1. Foreground selection in introgression lines in IC_4F_6 generation for targeted genes *xa13, Xa21, xa5, Pi54* and *Bph20* in the background of Krishna Hamsa. DP-Donor parent, RP-Recurrent parent.

Supplementary Table S1. Selection of polymorphic markers between recurrent parents and donors for foreground selection

Gene/QTL	Markers screened for FS	Selected Markers	References
xa5	RM122, RM159, RM13, RM17795, RM4777, xa5R, xa5S, RM13,	RM122	[58]
xa13	xa13 pro, RM6765, RM6070, SR6	xa13 pro	[14]
Xa21	pTA248, *Xa21*	pTA248	[59]
Pi2	RM 527	RM 527	[28]; [60]
Pi9	S29742c, S53395c, RM 136, RM7311, PI9STS2	PI9STS2	[59]
Pi54	PiKh MAS	PiKh MAS	[28]; [60]
Bph20	RM 28570, B42, B44	RM 28570	[31]
Bph21	RM 28491, S12094AF, RM2857OF, B122	RM 28491	[31]
qDTY1.1	RM11943, RM212, RM315, RM3825, RM12091, RM12023, RM431,	RM431, RM11943	[61]
qDTY3.1	RM15736, RM15738, RM15926, RM15924, RM16032, RM15914, RM15925, RM55, RM168, RM186, RM293, RM468, RM571, RM16030, RM16049, RM416, RM520	RM168	[37]
qDTY2.1	Hvssr2-31	HvSSR2-31	[62]
qDTY12.1	RM 511 , RM 28166,	RM511/28158, RM28166	[35]

FS- foreground selection

Supplementary Table S2 Analysis of Variance for days to fifty percent flowering and grain yield (g/m^2) traits under control conditions (*kharif* 2019)

Source of Variation	d.f	DFF			GY (g/m^2)		
		Mean Squares	F-Calculated	Significance	Mean Squares	F-Calculated	Significance
Blocks (eliminating	4	0.50	0.24	0.91	36910	2.12	0.13

treatments)							
Treatment (ignoring blocks)	199	65.17	31.79	0.00	122242	7.01	0.00
Control	4	78.30	38.20	0.00	13582	0.78	0.56
Treatments	194	64.37	31.41	0.00	124637	7.15	0.00
Control *Vs* Treatment	1	166.80	81.37	0.00	92278	5.29	0.04
Error	16	2.05			17437		
Total	219						

Treatment refers to 196 introgression lines, control to five checks in Augmented block design. d.f.- degrees of freedom, DFF- days to fifty percent flowering, GY- grain yield in g/m^2. F value indicates significant differences among the ILs and checks under non-stress conditions across different sources of variation except 'blocks' and 'control'.

Supplementary Table S3 Analysis of Variance for days to fifty percent flowering and grain yield (g/m^2) traits under reproductive stage drought stress conditions (*kharif* 2019)

Source of Variation	d.f.	DFF			GY (g/m^2)		
		Mean Squares	F-Calculated	Significance	Mean Squares	F-Calculated	Significance
Blocks (eliminating treatments)	4	30863	0.98	0.45	7726	1.11	0.39
Treatment (ignoring blocks)	101	1692	0.05	1.00	22713	3.25	0.01
Control	4	35508	1.13	0.38	93420	13.38	0.00
Treatments	96	44	0.00	1.00	15129	2.17	0.04
Control *Vs* Treatment	1	24602	0.78	0.39	467992	67.02	0.00
Error	16	31542			6983		
Total	121						

Treatment refers to 196 introgression lines, control to five checks in Augmented block design. d.f.- degrees of freedom, DFF- days to fifty percent flowering, GY- grain yield in g/m^2. F value indicates significant differences among the ILs and checks under reproductive stage drought stress conditions across different sources of variation except 'blocks' and 'treatment'.

Supplementary Table S4 Descriptive statistics and heritability for days to fifty percent flowering and grain yield (g/m^2) traits under control conditions (*kharif* 2019)

	Control		Reproductive stage drought stress	
	DFF	GY (g/m^2)	DFF	GY (g/m^2)
Mean	98.77	574	99.51	403
Standard Error	0.52	22.66	7.36	12.85
Standard Deviation	7.71	336.17	81.26	141.94
Range	32	2889	911	624
Minimum	86	108	76	98
Maximum	118	2998	106	722
**h^2(bs)**	96.81	86.00	49.96	70.73

DFF- days to fifty percent flowering, GY- grain yield in g/m^2.

Supplementary Table S5 Critical differences (CD) for days to fifty percent flowering and grain yield (g/m^2) traits under reproductive stage drought stress conditions (*kharif* 2019)

Comparison type	Control				Reproductive stage drought stress			
	DFF		GY (g/m^2)		DFF		GY (g/m^2)	
	C.D. at 5 %	C.D. at 1 %	C.D. at 5 %	C.D. at 1 %	C.D. at 5 %	C.D. at 1 %	C.D. at 5 %	C.D. at 1 %
Between two Control Mean	1.92	2.65	238	328	112	154	177	244
Between two treatments in same block	4.29	5.91	532	734	251	345	396	545
Between two treatments not in same block	4.70	6.48	583	804	274	378	434	598
Between treatment and control	3.64	5.02	452	622	213	293	336	463

DFF- days to fifty percent flowering, GY- grain yield in g/m^2, C.D. Critical difference

Supplementary Table S6 Phenotypic evaluation for yield under control conditions (*kharif* 2019)

Entry No	DFF	GY (g/m^2)
**PC I: BLB + blast + drought**		
19174	99	737
19193	97	374
19196	90	544
19246	93	561
19247	112	598
**PC II: BLB+ blast**		
19019	97	610
19030	92	1010
19471	93	543
19007	89	489
19020	98	716
19025	90	766
19031	88	872
19406	95	311
19039	88	644
19378	92	567
**PC III: BLB + drought**		
19232	99	550
19233	111	395
19238	94	600
19239	108	507
19240	109	565
19241	107	693
19244	110	418
19245	101	475
19248	113	628
**PC IV: Blast + drought**		
19176	105	603
19177	92	433
19178	99	570
19181	97	407
19182	99	341
19183	99	301
19185	92	1736
19189	93	501
19190	92	345
19191	99	721
19192	98	298
19194	100	446
19195	88	586
19197	92	243
19198	109	334
19199	110	402
19200	88	449
19201	92	666
19203	111	296
19204	114	343
19205	101	372
19206	92	330
19208	94	349
19211	101	1289
19214	98	552
19215	96	404
19221	107	858

Entry No	DFF	GY (g/m^2)
19237	97	642
19249	100	891
19250	112	522
19253	109	315
19254	111	180
19261	109	359
19262	109	368
19263	111	655
19264	112	567
19267	113	655
19268	108	342
19271	109	524
19273	100	875
19274	111	585
19275	93	413
19279	107	284
**PC V: BLB**		
19046	92	2244
19460	103	631
19379	118	350
**PC VI: Blast**		
19035	92	941
19004	93	915
19053	92	908
19013	87	850
19403	99	831
19048	92	823
19021	95	808
19005	92	800
19006	93	794
19056	92	761
19027	86	760
19024	88	757
19028	92	746
19026	112	744
19243	92	743
19001	108	737
19022	93	726
19413	88	712
19415	99	675
19402	98	649
19175	100	646
19023	93	628
19401	99	628
19018	110	588
19032	95	575
19421	101	564
19014	88	548
19050	96	547
19043	89	539
19015	96	522
19409	89	513
19044	89	506
19397	88	501
19470	92	498

Entry No	DFF	GY (g/m^2)
19466	98	496
19410	92	482
19468	101	469
19464	103	467
19052	89	449
19045	88	449
19210	89	434
19459	98	433
19180	92	433
19469	101	428
19408	93	427
19392	99	424
19387	93	415
19412	93	413
19463	103	395
19467	101	388
19462	99	385
19465	97	382
19386	99	372
19396	88	357
19411	92	353
19461	103	345
19049	97	342
19400	99	316
19033	95	315
19054	88	305
19008	92	303
19037	93	292
19420	101	284
19389	93	272
19038	92	264
19417	99	217
19399	89	216
19407	93	201
19416	101	197
19034	93	180
19394	88	141
19405	88	108
19167	88	147
19172	98	362
19186	89	206
19188	92	324
19202	109	1174
19207	113	606
19270	112	567
19272	111	2706
**PC VII: Drought**		
19209	99	1282
19218	87	1221
19213	100	1023

Entry No	DFF	GY (g/m^2)
19227	109	983
19231	101	957
19281	95	820
19242	93	818
19277	95	799
19219	102	786
19226	97	759
19280	88	746
19234	98	734
19228	106	722
19251	113	704
19255	111	684
19173	100	656
19257	110	654
19184	92	588
19217	88	582
19252	110	573
19265	113	565
19220	114	560
19230	99	551
19269	99	544
19235	97	528
19168	90	522
19216	95	522
19222	109	484
19229	100	481
19278	88	475
19166	92	462
19179	110	450
19169	90	449
19170	107	429
19187	90	417
19224	105	416
19236	96	407
19266	113	391
19223	108	373
19171	98	364
19259	112	336
19258	110	326
19225	108	282
Krishna Hamsa	98.4	486.4
MTU 1010	98.8	473.8
IR 64	99	575.6
Saha bhagidhan	102	476.4
IR81896	107.8	571.6

DFF- days to fifty percent flowering, GY- grain yield in g/m^2.

Supplementary Table S7 Phenotypic evaluation for yield under reproductive stage drought stress (*kharif* 2019)

Entry No	DFF	GY (g/m^2)
**PC I: BLB + blast + drought**		
19246	100	538

Entry No	DFF	GY (g/m^2)
19247	98	444
19196	76	423

Entry No	DFF	GY (g/m^2)
19174	95	350
19193	90	323

Entry No	DFF	GY (g/m²)
**PC III: BLB + drought**		
19233	95	638
19245	93	635
19248	101	452
19232	97	436
19240	87	433
19238	95	333
19241	91	307
19239	93	307
19244	99	251
19273	94	694
19198	98	691
19178	106	685
19254	95	618
19215	97	597
19274	97	581
19201	95	574
19177	85	560
19268	95	554
19262	90	535
19199	101	514
19267	102	508
19181	97	506
19264	91	490
19261	77	488
19195	77	487
19279	94	477
19275	94	467
19249	76	461
19211	99	451
19192	83	448
19263	100	440
19253	95	434
19221	93	412
19183	95	411
19214	98	410
19194	91	387
19250	87	381
19200	98	380
19189	81	351
19191	98	347
19208	85	332
19190	82	324
19176	90	324
19237	95	313
19203	76	299
19197	76	298
19271	92	293
19182	95	281
19206	87	273
19205	81	224
19185	83	221
**PC VII: Drought**		
19226	94	722
19278	94	660
19251	95	608
19231	96	605
19280	99	584
19234	95	582
19224	98	557
19277	94	547
19225	94	546
19219	95	523
19223	93	512
19222	93	508
19235	95	503
19173	95	489
19269	89	487
19281	94	479
19242	95	473
19266	101	472
19252	95	451
19216	95	451
19170	95	450
19259	90	439
19258	86	414
19236	95	408
19166	90	404
19228	99	390
19213	99	387
19218	95	368
19209	86	360
19169	83	353
19217	95	351
19257	87	344
19227	99	314
19184	91	287
19187	81	275
19220	99	254
19229	96	254
19171	83	248
19168	83	243
19255	95	238
19230	96	237
Krishna Hamsa	88	192
MTU 1010	88	238
IR 64	92	347
Sahabhagi dhan	89	144
IR81896	102	485

DFF- days to fifty percent flowering, GY- grain yield in g/m^2.

Supplementary Table S8. Polymorphic markers between pairs of parents for use in background selection

Chr. No.	Total No. of markers analyzed	Polymorphism between Krishna Hama and each of the donor parent<br>IRBB60	Tetep	DSB2	IR74371	IR81896	IR96321
1	44	RM3341<br>RM488<br>RM11377<br>RM212<br>(4)	RM10074<br>RM243<br>RM3627<br>RM10963<br>RM3341<br>RM11391<br>RM1244<br>RM11569<br>RM3825<br>RM11943<br>RM12276<br>(11)	RM594<br>RM11068<br>RM11191<br>RM3341<br>RM488<br>RM11377<br>RM11391<br>RM297<br>RM11943<br>(9)	RM10074<br>RM595<br>RM3341<br>RM488<br>RM11377<br>RM11943<br>RM12276<br>(7)	RM10074<br>RM3627<br>RM595<br>RM3341<br>RM488<br>RM11377<br>RM11943<br>(7)	RM10074<br>RM243<br>RM3627<br>RM595<br>RM3341<br>RM5853<br>RM2318<br>RM488<br>RM11377<br>RM11391<br>RM1117<br>RM297<br>RM3825<br>RM11943<br>(14)
2	34	RM6378<br>RM2468	RM12433<br>RM6378	RM12433<br>RM6378	RM3549<br>RM1234	RM12307<br>RM12434	RM13384<br>RM3515

Chr. No.	Total No. of markers analyzed	Polymorphism between Krishna Hama and each of the donor parent					
		IRBB60	Tetep	DSB2	IR74371	IR81896	IR96321
		RM3549<br>RM1234<br>RM3515<br>RM13607<br>RM1942<br>RM13863<br>(8)	RM3549<br>RM1234<br>RM3515<br>RM13607<br>RM1942<br>RM13863<br>(8)	RM2468<br>RM3549<br>RM6374<br>RM4499<br>RM13393<br>RM13607<br>RM1942<br>RM3316<br>RM208<br>(11)	RM6374<br>RM13384<br>RM13393<br>RM3515<br>RM13863<br>RM3316<br>(8)	RM279<br>RM6378<br>RM3549<br>RM13384<br>RM3515<br>RM208<br>(8)	RM13607<br>RM13863<br>RM3316<br>RM14170(6)
3	35	RM15223<br>RM8072<br>(2)	RM15488<br>RM15803<br>RM520<br>RM8072<br>(4)	RM8072<br>(1)	RM3350<br>RM520(2)	RM15191<br>RM16<br>RM15488<br>RM3350<br>RM520(5)	RM15488<br>RM520<br>RM8072<br>(3)
4	41	RM16591<br>RM16864<br>RM551<br>RM8213<br>RM 8072<br>(5)	RM16262<br>RM16591<br>RM17022<br>RM17104<br>RM17305<br>RM551<br>RM8213<br>RM 8072<br>(8)	RM16459<br>RM16591<br>RM16642<br>RM17022<br>RM8213<br>RM 8072<br>(6)	RM<br>80729(1)	RM16262<br>RM16591<br>RM17022<br>RM551<br>RM8213<br>(5)	RM8213<br>RM551<br>RM17022<br>RM16591<br>(4)
5	26	RM1248<br>RM17855<br>RM18394<br>RM3381<br>RM18933(5)	RM17855<br>RM18018<br>(2)	RM17855<br>RM18018<br>(2)	--	--	--
6	45	RM588<br>RM206<br>RM439<br>(3)	RM588<br>RM19642<br>(2)	RM589<br>RM19642<br>(2)	--	RM588<br>RM253<br>RM19642<br>RM18405<br>RM3437<br>RM206<br>RM19056(7)	RM588<br>RM253<br>RM19642<br>RM3437<br>RM206<br>RM19056(6)
7	37	RM20794<br>RM20896<br>RM21445<br>RM20917<br>(4)	RM 20794<br>RM20896<br>RM21445<br>RM22100<br>(4)	RM1135<br>RM21445<br>RM20917<br>(3)	RM1135<br>RM20794<br>RM 144<br>(3)	RM 144<br>RM20794<br>RM209179(3)	--
8	23	RM407<br>RM1235<br>(2)	RM 38<br>RM22551<br>RM22710<br>RM407<br>RM 447<br>(5)	RM22685<br>(1)	RM38<br>RM22709<br>RM22710<br>RM2251<br>RM515<br>(5)	RM515<br>RM22710<br>RM22709(3)	RM2251<br>RM22709<br>RM22710<br>RM515(4)
9	19	--	--	RM24715<br>RM7481<br>(2)	--	--	--
10	23	RM25187<br>RM6100<br>RM332<br>(3)	RM25124<br>RM25187<br>RM25658<br>RM25846<br>RM216<br>RM25581	RM24899<br>RM25187<br>RM25472<br>RM25846<br>RM6100<br>RM 216	--	RM24899<br>RM25124<br>RM6100<br>RM25581<br>(4)	RM25124<br>RM25472<br>RM332(3)

Chr. No.	Total No. of markers analyzed	Polymorphism between Krishna Hama and each of the donor parent IRBB60	Tetep	DSB2	IR74371	IR81896	IR96321
			RM332<br>(7)	RM 258<br>(7)			
11	25	RM206<br>RM26200<br>RM332<br>RM4112<br>(4)	RM224<br>RM26119<br>RM26200<br>RM27322<br>(4)	RM202<br>RM26200<br>RM26315<br>RM27322<br>RM332<br>RM27367<br>(6)	--	RM26200<br>RM27322<br>RM332<br>(3)	RM206<br>RM26200<br>RM27322<br>(3)
12	23	RM28199<br>RM28488<br>RM3331<br>RM28570<br>RM235<br>RM17<br>(6)	RM224<br>RM17<br>RM27289<br>(3)	RM28199<br>RM28357<br>RM28488<br>RM3331<br>(4)	--	RM7102<br>RM28357<br>RM28488<br>RM28570<br>RM28166<br>(5)	RM26200<br>RM27322<br>RM332<br>(3)
**Total**	**375**	**47**	**58**	**54**	**26**	**50**	**47**
